# MEF2 and the Right Ventricle: From Development to Disease

**DOI:** 10.3389/fcvm.2019.00029

**Published:** 2019-03-28

**Authors:** Katharine R. Clapham, Inderjit Singh, Isabella S. Capuano, Sudarshan Rajagopal, Hyung J. Chun

**Affiliations:** ^1^Section of Cardiovascular Medicine, Department of Internal Medicine, Yale Cardiovascular Research Center, Yale School of Medicine, New Haven, CT, United States; ^2^Section of Pulmonary, Critical Care and Sleep Medicine, Department of Internal Medicine, Yale University School of Medicine, New Haven, CT, United States; ^3^Choate Rosemary Hall, Wallingford, CT, United States; ^4^Division of Cardiology, Department of Medicine, Duke University Medical Center, Durham, NC, United States

**Keywords:** right ventricle, MEF2, pulmonary hypertension, HDAC, hypertrophy

## Abstract

Pulmonary arterial hypertension is a progressive and ultimately life-limiting disease in which survival is closely linked to right ventricular function. The right ventricle remains relatively understudied, as it is known to have key developmental and structural differences from the left ventricle. Here, we will highlight what is known about the right ventricle in normal physiology and in the disease state of pulmonary arterial hypertension. Specifically, we will explore the role of the family of MEF2 (myocyte enhancer factor 2) transcription factors in right ventricular development, its response to increased afterload, and in the endothelial dysfunction that characterizes pulmonary arterial hypertension. Finally, we will turn to review potentially novel therapeutic strategies targeting these pathways.

## Introduction

Pulmonary arterial hypertension (PAH) remains a significant therapeutic challenge, despite ongoing advances in our body of knowledge of this devastating disease. In particular, no approved therapies to date seek to directly achieve improvement or preservation of the right ventricular (RV) function, despite compelling evidence that RV function is a critical determinant of clinical outcomes in PAH. Recent studies have begun to investigate strategies to preserve or improve RV function in the context of increased pulmonary vascular resistance (PVR), including application of left-sided heart failure treatments to RV failure. However, the two ventricles are uniquely distinct in their embryonic origins, and multiple studies have demonstrated that even in adult stages their gene expression profile is remarkably different, suggesting that “one size fits both” approach to left ventricular (LV) and RV failure may face challenges in demonstrating tangible clinical efficacy. In this review we discuss what is known about mechanisms of RV development and disease, and highlight recent advances that may lead to novel RV-based therapeutic strategies.

## The Normal RV

In the fetal circulation, because of its extensive network of capillaries, the placenta has the lowest vascular resistance. In contrast, the PVR is high because of the fluid filled lungs. Consequently, two right to left shunts connecting the pulmonary and systemic circulations are encountered in the fetal circulation, namely the foramen ovale and the ductus arteriosus. The fetal RV is a thick-walled chamber that ejects blood into the high resistance pulmonary vascular system. Starting week 12 of gestation to term, the RV wall thickness is comparable to that of the LV. At birth, the pulmonary circulation regresses into a low resistance, high capacitance system, and the RV is able to eject directly into the pulmonary circulation without having to bypass the lung via foramen ovale or ductus arteriosus. The RV thickness subsequently regresses, leading to the thin-walled RV as seen in normal adults (1). Although the molecular mechanism that initiates this process is uncertain, an intriguing hypothesis involves the loss of fetal cardiomyocyte hypertrophic and proliferative genes allowing for normal RV morphologic adaptation to its pulmonary circulation.

The RV chamber is smaller and produces less contractile force than its counterpart. However, the RV is able to achieve a similar cardiac output with lower energy expenditure. How is this possible? One of the reasons lies in the orientation of the RV fibers, which is distinct from that of the LV. The RV free wall is made of superficial, circumferentially oriented fibers and deeper longitudinally oriented myocardial fibers, while the LV is made up primarily of circumferentially oriented fibers (1). At the end of diastole, the RV free wall is 2–3 mm in thickness while the LV free wall is 8 to 11 mm^2^. This thin longitudinal fiber orientation allows for a greater RV end-diastolic volume and surface area per volume of blood ensuring an identical stroke volume as the LV with lower energy expenditure (2). The longitudinal fiber orientation is also responsible for the peristaltic like RV contraction that begins at the inflow region before proceeding to the mid-wall and finally the RV outflow tract. The inflow region of the RV contracts ~25–50 milliseconds before the outflow region (3). This peristaltic wave of contraction dilates the proximal pulmonary artery, priming it to receive the ejected stroke volume thereby facilitating RV stroke volume. Continued ejection of RV stroke volume is further enhanced by the low resistance and high capacitance nature of the normal pulmonary circulation. The RV is able to eject before and well-beyond attainment of peak RV systolic pressure. In fact, ~60% of the RV stroke volume can be ejected after peak RV systolic pressure (4).

The fiber orientation of the RV also explains its contractile nature, which is characterized by longitudinal shortening of the septum and transverse motion of the free wall towards the interventricular septum during systole (1). The oblique contraction of the septal fibers generates an ejection fraction of ~60% (5, 6) and plays an important role in normal RV systolic function. However, contraction of the RV free wall, which generates an ejection fraction of ~30%, plays an integral part in preserving RV function when septal akinesia or hypokinesia is commonly encountered following cardiac surgery. Incidentally, the oblique myocardial fibers of the LV free wall are in continuum with the superficial circumferential fibers of the RV free wall and also contribute significantly to RV contraction and pulmonary blood flow (7, 8). This systolic ventricular dependence may explain in part why the placement of an LV assist device in patients with LV systolic dysfunction with associated mild pulmonary hypertension (PH) can often precipitate acute RV failure (9).

The structural differences between the ventricular chambers account for their differing adaptive response to increasing preload and afterload. The more muscular LV, with its circumferentially arrayed fibers, is able to withstand sudden increases in afterload, but is less adaptive to increases in preload. On the other hand, the RV is able to accommodate large increases in venous return that is typical of the physiological response to exercise. This is likely because the normal RV is able to retain its shape rather than stretch, allowing it to adapt to increasing preload. The highly compliant RV is also able to function normally for decades in pathogenic states of increased preload, such as that seen in left to right intracardiac shunt defects (1). However, in scenarios of increasing RV afterload encountered in PH, the RV falters.

## RV Adaptation to PAH

Although the initial insult in PAH implicates the pulmonary vasculature, the functional state, exercise capacity, and survival of patients with PAH is closely linked to RV function (10). RV ejection fraction and end diastolic volume measured by cardiac magnetic resonance imaging (cMRI) have been shown to be independent predictors of mortality in PAH irrespective of afterload, further emphasizing the significance of RV function as a prognostic factor in PAH (11).

RV adaptation to PAH is a complex process that not only relies on the severity of pulmonary vascular disease, but also on the interaction between neuro-hormonal activation, coronary perfusion, and myocardial metabolism (12, 13) (Table 1). Other factors that may influence RV adaptation include the underlying etiology of PAH, the age of onset, gender, ethnicity, as well as genetic and epigenetic factors (13). For example, males with PAH have worse RV function at initial presentation compared to their female counterparts (14), while the poorer outcomes observed in patients with scleroderma-associated PAH compared to idiopathic PAH may in part be related to the lower intrinsic RV contractility observed despite encountering similar RV afterload (15).

**Table 1:** Characteristics of an adaptive and maladaptive remodeling pattern of the right ventricle in pulmonary hypertension.

**Characteristic**	**Adaptive remodeling**	**Maladaptive remodeling**
**RV MORPHOLOGY**		
RV size	Normal size to mild dilatation	Enlarged RV
RV hypertrophy	Eccentric	Concentric
Mass-to-volume ratio	Higher	Lower
RV fibrosis	Absent	Present
**INTRINSIC RV FUNCTION**		
RV-PA coupling	Preserved or mild reduction	Reduced
RV contractile reserve	Preserved	Reduced
Resting RV ejection fraction	Normal to mild reduction	Reduced
RV-LV dyssynchrony	Present	Absent
RV perfusion with macro and microvascular ischemia	Normal or mild reduction perfusion Normal serum troponin	Reduced perfusion Elevated serum troponin
RV metabolism	Normal glucose uptake with normal mitochondrial oxidative phosphorylation	Increased glucose uptake with increase lactogenic glycolysis and dysregulated fatty acid oxidation
RV neuro-hormonal signaling	Preserved ß1 and DA receptor signaling Normal BNP	Desensitization/down regulation of ß1and DA receptor signaling Elevated BNP

*RV, right ventricle; PA, pulmonary artery; contractile reserve refers to ability of the RV to increase its contractility in the face of increasing afterload; LV, left ventricle; DA, dopaminergic*.

## Alterations in RV Geometry and Ventriculo-Arterial Coupling

The initial response of the RV to a chronic increase in afterload is to increase its contractile reserve to match the increasing afterload. This homeometric adaptation is achieved through RV myocyte hypertrophy leading to a concentric remodeling pattern, which is characterized by a higher mass-to-volume ratio that allows for preservation of RV contractile function (13). The matching of RV contractility (termed end-systolic elastance, Ees) and its afterload (termed arterial elastance, Ea) describes RV-PA coupling and its preservation allows for optimal RV functioning at minimal energy cost (16).

When the RV is no longer able to augment its contractile force in the face of increasing afterload, it shifts to rely on hetereometric or volumetric adaptation (i.e., the Frank-Starling mechanism) to sustain its flow output in response to increasing metabolic demand (17). The decreasing RV stroke volume leads to a compensatory increase in the heart rate, which in turn further increases the Ea (18). The resulting increase in Ea with failure to mount a corresponding increase in Ees leads to RV-PA uncoupling seen in advanced stages of the disease and dynamically during exercise (17, 19). This maladaptive remodeling pattern is characterized by more eccentric hypertrophy and worsening RV systolic and diastolic function (10). RV volumetric adaptation stretches the tricuspid valve annulus, further decreasing forward flow, hampering LV filling, and further increasing the RV end-diastolic volume of an already pressure-loaded RV.

Despite the augmented susceptibility of the RV to increasing afterload, fibrosis of a chronically pressure overload RV occurs less extensively compared with a pressure-overloaded LV (e.g., systemic hypertension or severe aortic stenosis) (10). The fibrosis is limited to the RV-septal insertion points and this may explain why patients with severe pulmonary hypertension (PH) are able to attain normal RV function following lung transplantation, despite having reduced RV function at the time of transplantation.

## Alterations in RV Metabolism

The normal fetal RV is reliant on glycolysis and subsequent oxidative phosphorylation for ATP generation. However, in the normal adult RV, fatty acid oxidation becomes the primary mechanism for ATP generation for the RV and is responsible for ~60–90% of energy production in cardiomyocytes (1). In contrast to the normal RV, which is able to alternate its substrate use from fatty acids to glucose as needed, the myocytes of a hypertrophied RV exhibit suppression of mitochondrial oxidative phosphorylation, dysregulated fatty acid oxidation, and glutaminolysis (1, 13). Thus, the primary source of fuel is dependent on lactogenic glycolysis as opposed to fatty acid or glucose oxidation. This shift from oxidative phosphorylation to lactogenic glycolysis, known as the Warburg effect, is frequently observed in oncogenesis. This phenomenon is also seen in pulmonary vascular cells and in the failing RV in PAH patients. Hypoxia inducible factor (HIF) 1α has been identified as a main regulator of the Warburg effect. It is possible that in the presence of increasing RV afterload and therefore compromised coronary perfusion, the cellular oxygen tension is reduced. Consequently, HIF-1α is activated, leading to increased glucose uptake, increased expression glycolytic enzymes, decreased oxidative phosphorylation, increased lactate fermentation, and increased mitochondrial autophagy. Such reprogramming also leads to reduction in fatty acid oxidation (20).

Investigative studies have clearly demonstrated the importance of RV function as a major determinant of survival in patients with PAH. Future studies need to incorporate hemodynamic, metabolomic, and imaging phenotyping to improve our diagnostic approach allowing for a precision medicine strategy with RV targeted therapies tailored to individual patients.

## RV Development

As delineated above, the RV is unique in its structure and adaptive response to changes in physiology. In understanding the singular properties of the RV, it is illuminating to return to cardiac development. Initial descriptions of cardiac development were drawn from observations made of avian embryos, but subsequent study has demonstrated a broad applicability of these findings across species (21). Cardiac precursor cells from the mesoderm migrate to form the bilateral heart fields in chicks or cardiac crescent in mice, and the coalescence of these cells leads to the formation of a linear heart tube (22, 23). The heart field has been historically divided into the primary and anterior heart fields, with the primary heart field differentiating earlier and giving rise to the inflow limb, and the anterior heart field forming the outflow limb (22). However, subsequent clonal analysis has given rise to a new classification system in which the cells of the first heart field (FHF) are recognized to be the origin of the LV, part of the RV, and atria, and the second heart field (SHF) forms the majority of the RV, atria, and outflow tract (22). This classification system follows *Isl1* expression to some degree, although more recent studies have indicated that *Isl1* is expressed in both heart fields (24–28). The heart tube subsequently undergoes rightward looping, forming and orienting the outflow tract relative to the future chambers of the heart, before the formation of the atrioventricular valves and cardiac chambers (23).

This complex series of events is orchestrated by a genetic program controlling cell fate. The earliest recognized marker of cardiac cells arising from the mesoderm is the transcription factor *Mesp1*, though *Mesp1* labeled cells give rise to other mesoderm derived cell populations as well (29). Myocardial cell differentiation begins to occur in the cardiac crescent, and the SHF phenotype is driven by a regulatory network which includes ISLET1 and NKX2-5 (30). The anterior part of the SHF is characterized by expression of TBX1, FGF8, or FGF10 and activation of the *Mef2c* enhancer, and gives rise to the RV and the outflow tract (30).

## MEF2 in Cardiac Development

*Mef2c* (Myocyte enhancer factor 2C), one of four members of the MEF2 family of transcription factors in vertebrates, is thus critical to the development of the RV and outflow tract. The members of the MEF2 family of MADS (MCM1, agamous, deficiens, SRF)-box transcription factors have a broad range of functions as transcriptional activators to govern pathways leading to cell differentiation, proliferation, and survival in a variety of cell types (31). The highest expression of *Mef2* genes is in the striated muscles and brain, although MEF2 is expressed in most tissues, including in smooth muscle and endothelium (32, 33). MEF2 serves a key role in vertebrate skeletal muscle differentiation, heart, neural crest, bone, vascular, and T cell development, and acts in adult tissues as a mediator of stress-induced remodeling (31).

The function of MEF2 in vertebrate cardiac development has been explored through the deletion and overexpression of *Mef2* family members, demonstrating that MEF2 is required for cardiomyocyte differentiation and cardiac organogenesis. *Mef2c* is the first of the *Mef2* genes to be expressed in mouse and chick embryos, initially in the mesoderm giving rise to the heart (32). The hearts of mice homozygous for a null mutation of *Mef2c* failed to undergo looping morphogenesis and develop a right ventricle (34). Cardiac overexpression of a dominant negative MEF2C protein inhibits cardiomyocyte differentiation, mediated in part by decreased GATA4 and NKX2-5 expression (35). *Mef2a* null mice are prone to sudden death in the perinatal period, and demonstrate cardiomyocyte myofibrillar disorganization followed by pronounced RV dilation (36). *Mef2b* and *Mef2d* null mice demonstrate no developmental cardiac defects (37, 38).

The identification of abnormal MEF2 activity in individuals with congenital heart disease reinforces the relevance of the animal findings to human cardiac development. Screening of a patient population with congenital heart disease for mutations in candidate genes demonstrated a heterozygous missense *MEF2C* mutation leading to decreased transcriptional activity in a family with double outlet RV and ventricular septal defect (39). Another study of 256 patients with cardiac outflow tract defects led to the identification of a sequence variant in *MEF2C*, which perturbed cardiac development in zebrafish when overexpressed (40).

## MEF2 in Cardiac Hypertrophy

While it is clear that MEF2 has a critical role in cardiac development, there is a body of literature to suggest its role in cardiac hypertrophy. In animal models, MEF2 DNA binding activity is at its peak in the late fetal and neonatal heart, declines to low levels in adulthood, but is increased in the setting of volume or pressure overload (41). *Mef2c* expression and MEF2C protein levels are increased in the myocardium of mice with angiotensin II induced cardiac hypertrophy, and MEF2C silencing by siRNA attenuates ventricular hypertrophy in an angiotensin II and aortic banding model of cardiac hypertrophy (42, 43). Transgenic mice with overexpression of MEF2A and MEF2C demonstrate increased ventricular weight, but decreased ventricular contractility; overexpression of MEF2D also causes pathologic cardiac remodeling (37, 44). Hypertrophic stimuli are thought to increase MEF2 activity through multiple mechanisms, including CaMK (Ca^2+^/calmodulin-dependent protein kinase) mediated nuclear export of inhibitory class IIa HDACS (HDAC4 and 5), MAPK induced phosphorylation of MEF2, and calcineurin signaling (45–50). Expression of a dominant negative form of MEF2 is also able to normalize the cardiac hypertrophy seen in calcineurin transgenic mice (51). Class IIa HDACs therefore act a “calcium sensitive” switch for cardiac hypertrophy through regulation of MEF2 (52).

While available evidence links MEF2 activity to LV hypertrophy, important questions remain in the context of RV function and adaptation to PH. First, the role of MEF2 in the transition between compensatory LV hypertrophy and subsequent dilation and decompensation remains inadequately defined—it is possible that MEF2 may be required to drive the initial hypertrophic response, but subsequent decompensation and worsening of ventricular function may involve other signals. Second, the studies of MEF2 in cardiac hypertrophy have focused predominantly on the LV, with a relative dearth of information about the function of MEF2 in the RV response to increased afterload in PAH. This deficiency of evidence stems in part from the use of genetic tools that lack ventricular specificity. While *Mef2c*-AHF-Cre transgenic mice have been utilized to label cells that derive from the second heart field to provide insight into cardiac development (53), similar approaches have not yet been undertaken in the investigation of the RV specific molecular mechanisms of disease.

**Figure 1:**
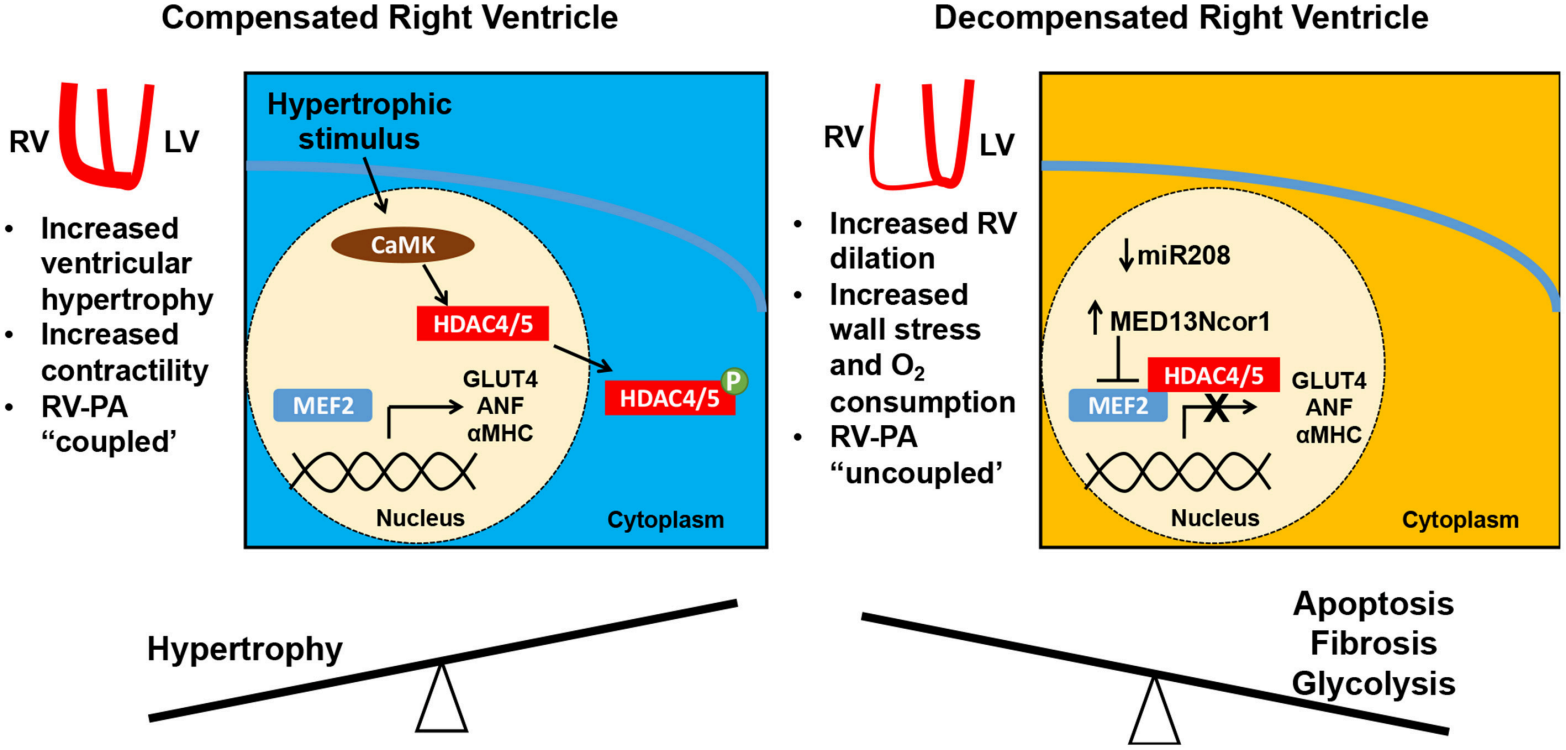
Right ventricular (RV) adaptation in pulmonary hypertension. In response to increasing pulmonary arterial (PA) pressure, the RV hypertrophies and augments its contractility (RV-PA coupling is maintained). This adaptive RV hypertrophic response is mediated in part by MEF2C with expression of MEF2 targets such as GLUT4, ANF, and aMHC. However, with a progressive increase in RV afterload the RV dilates, increasing the free wall stress and myocardial oxygen consumption. Consequently, the RV-PA unit is uncoupled. During this decompensated phase, there is a decrease in MEF2C levels, which is linked to mir208 suppression and increased MED13/NCoR1 activity. NCoR1 is known to decrease *Mef2c* transcription and acts in a complex with HDAC3/4 to deacetylate MEF2D and decrease its activity. MEF2C, myocyte enhancer factor 2C; miR, microRNA.

A recent study has provided some insight into how MEF2 guides the RV response to increased afterload. As reviewed above, initially in PAH, the RV hypertrophies in a compensatory fashion, but ultimately decompensation and decline of RV function can follow. In a monocrotaline rat model of PH, MEF2C levels and nuclear localization increase in the RV in the first several weeks of compensatory RV hypertrophy, then decrease with RV decompensation (54). Downregulation of MEF2C in decompensated RV hypertrophy occurs through suppression of microRNA 208 (miR-208) and increased MED13/NCoR1 activity, a pathway also upregulated in the RV in response to the proinflammatory stimulus of TNF-α (54). MEF2 activity is known in other contexts to be regulated at transcriptional, post-transcriptional and post-translational levels, and while comparatively little is understood of MEF2 regulation in the setting of RV hypertrophy and decompensation, transcriptional levels of *Mef2c* and *Mef2d* are known to be increased in the absence of NCoR1, and NCoR1 in conjunction with HDAC3 and HDAC4 has been demonstrated to lead to post-translational regulation of MEF2D by altering its acetylation state (55). MicroRNA profiling of the RV in mice subjected to pulmonary artery constriction demonstrates initial upregulation of microRNAs, such as miR-208, promoting cardiac hypertrophy and cardiomyocyte survival, and an increase in proapoptotic and fibrotic microRNAs in the decompensated stage (56). Taken together, the available evidence suggests that MEF2C may initially serve to promote an adaptive hypertrophic response to increased afterload in the RV, but that there is a shift to proapoptotic, fibrotic, and glycolytic pathways during decompensation that is associated with decreased MEF2 activity (56, 57) (Figure 1). This shift raises the question of whether there may be increased activity or nuclear localization of HDAC 4 and 5 in the decompensated RV, though this hypothesis has not yet been examined. The decreased MEF2 activity observed in the decompensated RV is in contrast to observations of the linkage between increased MEF2 activity and pathologic remodeling in the LV. This discrepancy may point to an interventricular difference in function and pathology, and warrants further investigation.

## MEF2 in the Endothelium

**Figure 2:**
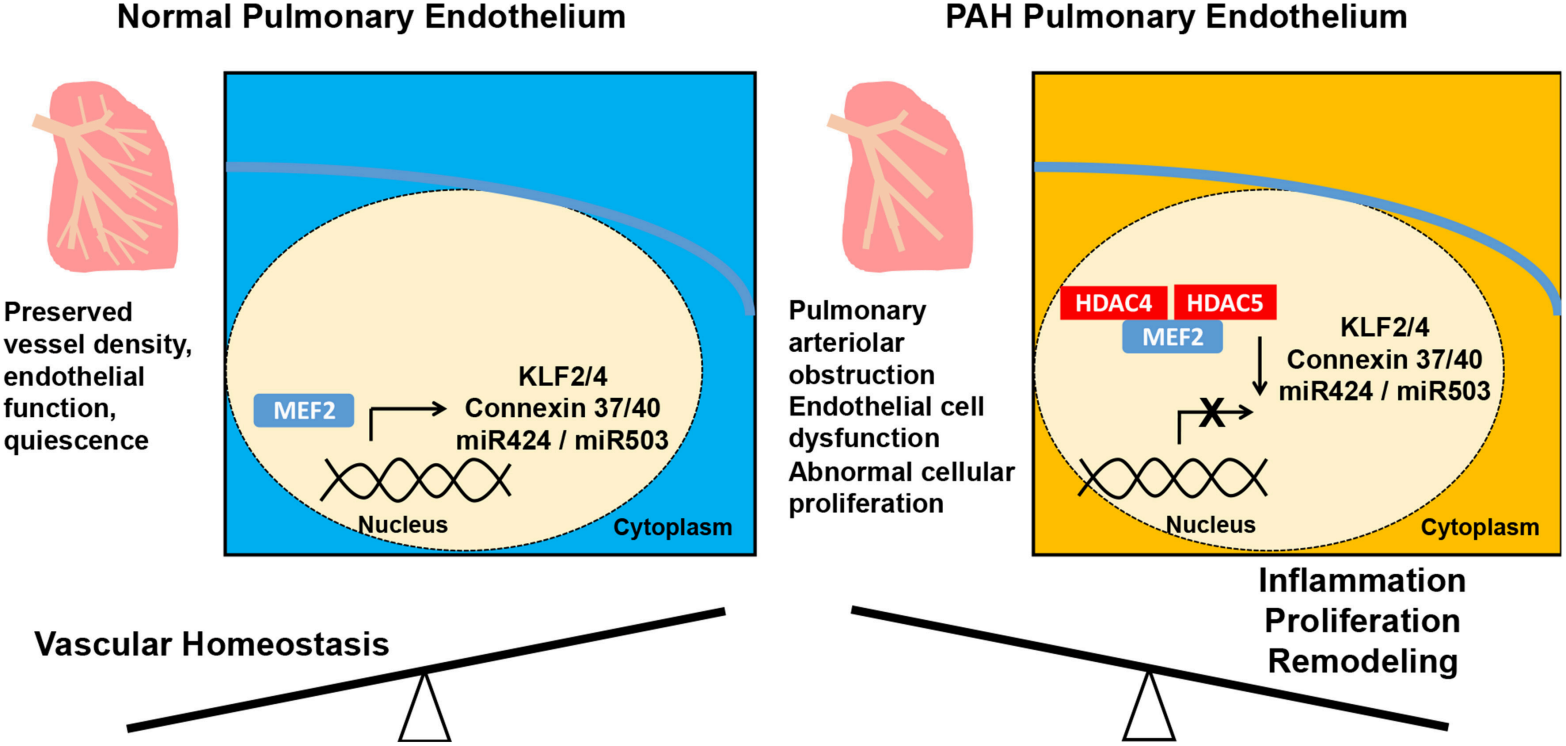
Alteration of the pulmonary vascular endothelium in pulmonary arterial hypertension (PAH). In the normal pulmonary vasculature, MEF2 promotes endothelial function preserving vessel density. In PAH, downregulation of MEF2 from increased nuclear localization of HDAC4 and HDAC5 results in decreased MEF2 activity and decreased expression of antiproliferative miR-424 and miR-503, the vascular homeostatic factors connexins 37, 40, and the anti-inflammatory and antithrombotic KLF2 and KLF4. These molecular alterations then lead to abnormal endothelial cell proliferation and muscularization of the pulmonary arterioles. MEF2, myocyte enhancer factor; HDAC, histone deacetylase; mir, micro ribonucleic acid.

PAH is of course a disease of the pulmonary vasculature as well, and MEF2 appears to have a protective role in the endothelium (Figure 2). MEF2A and MEF2C are the MEF2 isoforms most highly expressed in endothelium (58). MEF2C promotes endothelial survival, regulates endothelial cytoskeleton and inhibits smooth muscle migration into the intima of the vasculature (59, 60). Endothelial specific knock out of *Mef2c* in mice with subsequent exposure to hypoxia leads to increased PH, RV hypertrophy (RVH), and muscularization of pulmonary arterioles (61). The transcriptional activity of MEF2C is impaired in PAECs from individuals with PAH due to increased nuclear localization of inhibitory class IIa HDACs HDAC4 and HDAC5 (58). Decreased MEF2 activity leads to reduction in apelin mediated expression of miR-424 and miR-503, which decrease the pathologic hyperproliferation of endothelial and smooth muscle cells seen in PAH through downregulation of FGF2 and FGFR1 (58, 62). Reduced MEF2 activity also decreases the transcription of vascular homeostatic factors connexins 37, 40, and the anti-inflammatory and antithrombotic KLF2 and KLF4 (58, 63–67). Augmentation of MEF2 effects through class IIa HDAC inhibition decreases RVH and right ventricular systolic pressure (RVSP) in monocrotaline and Sugen/hypoxia rat models of PH (58, 61). In summary, MEF2C increases expression of multiple factors that maintain pulmonary vascular homeostasis, MEF2C activity is impaired in PAH, and class IIa HDAC inhibition can restore its function.

## HDAC IIa Inhibition Increases MEF2 Activity

MEF2, with its apparent protective role in RV function and endothelium in models of PAH, presents an attractive target for therapeutic intervention. As described above, class IIa HDACs bind MEF2 and suppress its activity (49, 50). Thus, HDAC inhibitors which would free MEF2 and increase transcription of its targets are of interest as potential disease-modifying agents in PAH.

Both pan HDAC and class specific HDAC inhibitors have been studied in preclinical models of PH (68). Surprisingly, the pan HDAC inhibitor tricostatin A (TSA), in a pulmonary artery banding (PAB) model of PH in Sprague Dawley (SD) rats leads to worse RV function and increased fibrosis, perhaps secondary to antiangiogenic effects of TSA (69). In contrast, class I HDAC inhibitor valproic acid does not lead to cardiac fibrosis or decreased capillary density in the same model (69). Administration of benzamide class I HDAC inhibitors to SD rats with hypoxia induced PH results in decreased PH with preserved RV function, but has a mild effect on RVH (70).

Notably, class II HDACs, especially class IIb HDAC HDAC6, were shown to increase VEGF expression in mouse cardiac microvascular endothelial cells, raising a concern that class II HDAC inhibition could have anti-angiogenic and deleterious effects similar to those of TSA (69). However, preclinical studies of class IIa HDAC inhibitors, increasing MEF2 activity, have had promising results. The class IIa HDAC inhibitor MC1568, when administered to rats with monocrotaline and Sugen/hypoxia induced PH, decreases RVH, RVSP, and muscularized arterioles without evidence of increased myocardial fibrosis or RV dilatation (58). Apoptosis, as measured by caspase 3 cleavage, is increased in human coronary artery endothelial cells with TSA but not MC1568 administration (58). Class IIa HDAC inhibitors TMP269 and Tasquinimod lead to a reduction in RVSP, RVH, and muscularized arterioles in a Sugen/hypoxia rat PH model (61). Whether the decrease in RVH despite increased MEF2 activity in these studies was due to decreased afterload or another mechanism remains to be further investigated.

## Conclusion

RV function is crucial to the outcome of patients with PAH. Understanding the RV's distinct development, structure, and adaptation to changing physiology is key to the development of appropriate management strategies and disease-modifying agents in PAH. MEF2, with its protective role in the face of increased afterload and endothelial dysfunction, is a prime candidate for such interventions. HDAC inhibitors, in particular class IIa HDAC inhibitors that appear to avoid the adverse effects noted with other classes, offer promise as a novel therapeutic strategy in a disease that has proven difficult to treat.

## Author Contributions

KC, IS, IC, SR, and HC devised the topic, wrote the manuscript, and prepared the figures.

### Conflict of Interest Statement

The authors declare that the research was conducted in the absence of any commercial or financial relationships that could be construed as a potential conflict of interest.
